# Advent of digital radiography: Part 1

**DOI:** 10.4103/0971-3026.40289

**Published:** 2008-05

**Authors:** BS Verma, IK Indrajit

**Affiliations:** Department of Radiodiagnosis and Imaging, Army Hospital (Research and Referral), Delhi Cantt. - 110 010, India

**Keywords:** Computed radiography, CR, digital radiography, direct digital radiography, DR, film-screen radiography, flat panel detectors, FSR, MTF, DQE, fill factor, pixel pitch, PSP plates

## Introduction

### Analog versus digital

In analog systems, a variable is measured on a continuous scale with an infinite number of possible values. In digital systems, however, measurements can only have a limited number of discrete values.[1] Illustratively, analog systems can be represented by an escalator ride where a person can be at any position from top to the bottom. Digital systems an be represented by a staircase where one can be only at a limited number of discrete positions.

Rapid advancement in the field of medical imaging has been possible due to the use of computers as they can process digital data very fast and efficiently. However, nature uses analog system including signals generated in diagnostic imaging. The human eye-brain system can handle analog signals very effectively. To use computers in medical imaging, analog data first need to be converted to digital data for processing and then converted back to analog images for viewing and interpretation.[2] This is done by analog-to-digital converters (ADC) and digital-to-analog converters (DAC), respectively. Most of the imaging devices in a radiology department, e.g. ultrasound, CT, MRI, DSA, etc., already use digital imaging technology.

## Radiography

Radiography is recording of information about an object using X-ray transmission. The intensity of X-rays is nearly uniform before entering an object being radiographed. After passing through the object, the spatial distribution of transmitted X-ray intensities carries all the radiographic information about the object. This information can be detected by means of something that is sensitive to radiation. Conventionally this is done by film-screen radiography (FSR). It can also be done by some digital detectors. When digital detectors are used to capture this information, the process is termed as digital radiography.

### Simplified definitions of some frequently used terms

As we have seen above, the spatial distribution of transmitted X-ray intensities carries all the radiographic information about the object. How faithfully and accurately this information is recorded is called the modulation transfer function (MTF). Thus, equipment with higher MTF will provide better spatial resolution. The efficiency with which this radiation information is captured is known as detective quantum efficiency (DQE).[3,4] Detectors with higher DQE will require less radiation dose than the detectors with lower DQE for similar image quality or signal-to-noise ratio (SNR).[5] Alternatively higher DQE detectors will provide better SNR for the same radiation dose.[5] Both MTF and DQE are depicted in the form of a graph as a function of frequency or spatial resolution in line pairs/mm (lp/mm). Both are higher at low resolution and decrease with increasing spatial resolution.[4] Most of the technical literature describes DQE at a spatial resolution of 0 lp/mm. Both DQE and MTF are higher in better detectors. DQE is a better and more comprehensive measure of the detector quality.

A digital detector has a large number of picture elements or pixels. All pixels are square in shape and “pixel size” is the length of one side in μm (micrometer). The distance between the centers of two adjacent pixels is known as the “pixel pitch”. As the distance between adjacent pixels is usually negligible, pixel pitch and pixel size are usually equal. Pixel size is a measure of limiting resolution, which is variously described as pixel size/pixel pitch in μm, pixels/mm, and lp/mm. Thus, a detector with a 200-μm pixel size may have a limiting spatial resolution depicted as 05 pixels/mm or 2.5 lp/mm.[6]

All the parts of a digital detector being exposed to radiation may or may not be able to convert X-rays into electrical signal. The area of the detector that is sensitive to X-rays in relation to the total detector area is known as the “fill factor”.[3] Detectors with higher fill factors are more efficient users of absorbed radiation.

## Conventional Radiography

In FSR, the absorbed X-rays are first converted into light by a pair of intensifying screens. Film sandwiched between these screens records a latent image that becomes visible after chemical processing. During the more than 100 years of its use, conventional radiography has been found to be very useful. Intensifying screens, introduced over 60 years ago and rare earth screens in recent years, have greatly reduced the radiation dose required for producing good quality images. Advancements in FSR technology have almost reached the limit of possible improvements. Only a completely new technology will be able to provide substantial advantage over the current FSR techniques. The advantages and limitations of FSR are listed in Table 1.

**Table 1:** Advantages and limitations of film-screen radiography

Advantages	Limitations
High spatial resolution Radiologist possesses a thorough knowledge of the entire imaging process to correctly pinpoint the cause of poor film quality Consistency of image appearance Familiarity and long experience leading to higher comfort level and confidence Acceptable film quality is only possible within narrow exposure limits; Discourages the use of excessive radiographic exposure	Acquisition, display, and storage of image are non-separable Limited exposure latitude resulting in frequent under- and overexposure of films[4] Chemical processing of films is essential.[4] Processing-related artifacts are common. Environment pollution by processing chemicals is of concern The process is inefficient as it is time and labor-intensive Single copy of film(s) is the end result. The film must be physically transported for viewing by different people involved in diagnosis/patient care[4] Fixed image contrast and density Only limited magnification of the image is possible with the use of magnifying lenses Relatively small size suitable for viewing by few people only. Needs to be converted to digital format to show larger audiences Film quality deterioration with time, especially if chemical processing is suboptimal Incompatibility with electronic imaging. Moreover, “filmless” department is not possible till the FSR is replaced by a digital method

## Digital Radiography Systems

A digital detector replaces film and screens in digital radiography. There are two basic types of digital radiography systems depending upon the types of detectors used to capture radiographic information:[7]

1. Computed radiography (CR) systems use a photo-stimulable phosphor (PSP) plate enclosed in a light tight cassette.[8] CR utilizes a two-stage process with the image capture and image readout done separately.
2. Direct Digital Radiography (DR) systems use detectors that have a combined image capture and image readout process.[4,7]

About two-thirds of patients visiting radiology departments are referred for plain radiography.[9] It is inevitable that conventional FSR will sooner or later be replaced by Digital Radiography due to the numerous advantages and electronic compatibility of the latter.

The advantages and limitations of the digital radiography systems (both CR and DR) are listed in Table 2.

**Table 2:** Advantages and limitations of digital radiography systems (both CR and DR)

Advantages	Limitations
Separation of acquisition, display, and archiving permits optimization of each activity separately[10] Wide exposure latitude with linear dose-signal relationship[11] Post-processing such as windowing, edge and contrast enhancement, magnification, direct measurements, cropping, annotation, etc. possible Reduced recall rates/repeat studies due to improper exposure[4,12] Simultaneous availability of images at different places Multiple exact copies can be made available. Teaching files can be created “Stitching” of adjacent images is possible with software to show long anatomical regions, e.g., full-length lower limbs, entire spine for scoliosis Compatible with teleradiology Computer-aided detection (CAD) can be used[4] Amenable to electronic archival and retrieval[4] Soft copy reporting can save cost of films	Radiation dose and film density relation is no longer valid. Lower dose produces images with more noise. Optimal as well as higher radiation dose produces good images.[13] To avoid noisy images, technicians have a tendency to use higher dose than necessary. This is called dose/exposure creep Inappropriate image enhancement may mimic disease Image appearance across different vendors is not consistent, especially with CR True size measurement of an object is difficult even when a scale is included in the film High cost of equipment, especially DR systems and the monitors suitable for soft copy reporting

## Computed Radiography Systems

CR cassettes use PSP plates in place of film and screens. These plates are coated with europium-activated barium fluoro-halide (BaFX: Eu^2+^).[14] The halide used may be bromide, iodide, or a combination of both. CR cassettes are used just like conventional cassettes on normal radiographic equipment and are available in similar sizes.

**Figure 1:**
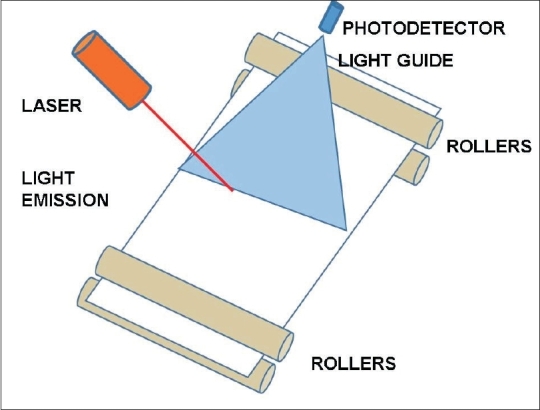
Schematic mechanism of a CR system. Imaging plate IP coated with photo-stimulable phosphor (PSP) is exposed to X-rays and records image data. Imaging plate within a cassette is taken to a CR reader, where it is scanned by a laser beam, which is swept across the plate by a rotating polygonal mirror. The light emitted from the imaging plate is collected by a fiber-optic bundle, converted to electric signal and used to form image

X-ray information is stored in PSP imaging plates as electrons, in semi-stable higher energy states, in sinks or “F” centers. The number of such trapped electrons is directly proportional to the absorbed X-ray dose. The imaging plate comes out or is exposed by opening the CR cassette within the CR reader. Image information is acquired by scanning the plate by a laser beam [Figure 1]. Red laser light excites these trapped electrons during scanning. Electrons eject from the higher energy sinks and come down to the base level. They emit a higher energy blue light during this process. This light is captured by a light guide, converted into electrical signals, amplified, digitized and used to form the image. The imaging plate is ready for reuse after exposure to white light.[15]

**Figure 2:**
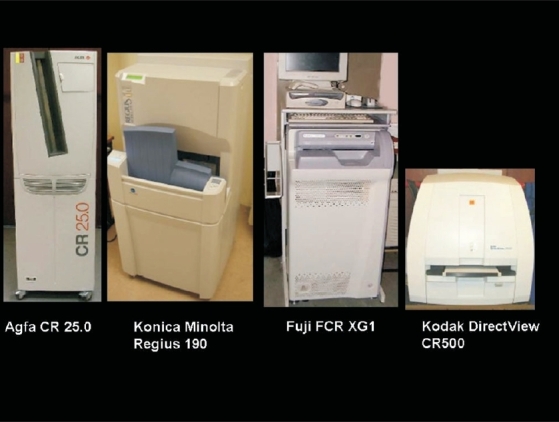
A pictorial mosaic of commonly available CR systems

Patient information and cassette ID needs to be linked in a CR system [Figure 2], as there is no direct electrical connection between the CR reader and the cassette. A bar code reader or a chip embedded on the CR cassette is used for this purpose. The PSP imaging plates may be flexible or rigid. The base used in these plates may be opaque or translucent. Due to different types of CR cassette designs and image readers available, all cassettes from the same vendor may not be compatible with all readers. Some of the CR plate readers can process one plate while holding multiple cassettes in a queue. This “drop and go” feature helps improve workflow.

Dual-side readout is available in some systems using PSP plates with translucent bases.[16] These systems use laser scanning from one side but capture light from both sides of the plate, increasing the DQE by 50 to 100%.[17] The spatial resolution of the CR images depends on the laser spot size, PSP plate characteristics (like packing density and thickness of the phosphor layer) and the sampling rate of the emitted light. Diffusion of the scanning laser light as well as the emitted light leads to some loss of spatial resolution. It is possible to achieve a resolution of 5-10 pixel/mm in general purpose CR cassettes. A resolution of 20 pixel/mm is available in most CR systems approved for mammography.

The time taken for scanning a PSP plate depends on the plate size, resolution desired, dual/single side readout and varies from 40 to 90 s. Some newly introduced systems use line scanning techniques, reducing the image read time to 20-30 s or even less. The advantages and limitations of CR systems are listed in Table 3.

**Table 3:** Advantages and limitations of computed radiography

Advantages	Limitations
Existing X-ray equipment can be used Single CR system can convert multiple radiography rooms to digital technology Great positioning flexibility for difficult views as CR cassettes can be placed in any position[8] Multiple cassette sizes available Cost effective route to digital radiography	The technique is time and labor-intensive like FSR Image reader takes time before the image can be displayed. Time taken is comparable to that required for film processing Speed class of 100-200 is similar to that of medium-speed film-screen system; Radiation dose required is same or more than FSR CR DICOM header carries less complete information

## Direct Digital Radiography Systems

To increase the workflow, it is important to avoid handling of the cassette, which is used in both, FSR and CR. This became possible with the availability of a new class of detectors, that were able to combine the processes of image capture and image readout, “without user intervention”.[15] Details of direct digital radiography systems and the effect of digital radiography technology on the projection radiography workflow will be covered in Part II of this article.
